# Fatigue and Durability of Laminated Carbon Fibre Reinforced Polymer Straps for Bridge Suspenders

**DOI:** 10.3390/polym10020169

**Published:** 2018-02-10

**Authors:** Fabio Baschnagel, Rea Härdi, Zafiris Triantafyllidis, Urs Meier, Giovanni Pietro Terrasi

**Affiliations:** 1 Mechanical Systems Engineering Laboratory, Empa, Swiss Federal Laboratories for Materials Science and Technology, Ueberlandstrasse 129, 8600 Duebendorf, Switzerland; fabio.baschnagel@empa.ch (F.B.); haerdire@student.ethz.ch (R.H.); 2 Department of Materials, ETH Zuerich, 8093 Zuerich, Switzerland; 3 Institute for Infrastructure and Environment, School of Engineering, The University of Edinburgh, Edinburgh EH9 3FG, UK; z.triantafyllidis@ed.ac.uk; 4 Empa, Swiss Federal Laboratories for Materials Science and Technology, Ueberlandstrasse 129, 8600 Duebendorf, Switzerland; urs.meier@empa.ch

**Keywords:** carbon fibre reinforced polymer, CFRP, fatigue, tensile elements, bridge suspenders

## Abstract

Steel cables and suspenders in bridges are at high risk of corrosion-fatigue and in some cases of fretting-fatigue in their anchorages. These factors greatly limit the service stresses of a specific cable system and involve expensive protection measures. In order to investigate the above limitations, the fretting fatigue behaviour of pin-loaded carbon fibre reinforced polymer (CFRP) straps was studied as models for corrosion-resistant bridge suspenders. Two types of straps were tested: small model straps with a sacrificial CFRP ply and large full-scale straps. In a first phase, five fully laminated and carbon pin-loaded CFRP model straps were subjected to an ultimate tensile strength test. Thereafter, and in order to assess their durability, 20 model straps were subjected to a fretting fatigue test, which was successfully passed by 4 straps. An S-N curve was generated for a load ratio of 0.1 and a frequency of 10 Hz. In a second phase, one full-scale strap was tested for its ultimate tensile strength and two full-scale straps were fatigue-tested. The influence of fretting fatigue loading on the residual mechanical properties of the straps was also assessed, and although fretting fatigue represented an important limitation for laminated CFRP straps, it could be shown that the investigated CFRP tension members can compete with the well-established steel suspenders.

## 1. Introduction

Carbon fibre reinforced polymers (CFRP) have attracted significant attention in the past few decades as alternative materials to steel for tendons and suspension cables due to their superior strength and stiffness, low weight, and excellent resistance to corrosion, fatigue, and creep. Despite these favourable characteristics of CFRP materials, their widespread implementation was partially hindered by significant challenges regarding their anchorage to structural elements (mainly due to the anisotropic nature of CFRP as opposed to steel). Although simple approaches, such as clamping devices for quasi-static tensile loads, have been developed in the recent past [1,2], the anchorage of fatigue loads poses a serious limitation to such clamping anchorage systems. Thus, complex anchoring systems are required to ensure full exploitation of the tendon properties under fatigue loads [1,3]. To overcome this complexity, pin-loaded looped CFRP straps have been suggested by Meier and Winistoerfer [4] as practical tensile elements that are stressed by load transfer through the pins without the need for additional anchoring systems. Nowadays, pin-loaded unidirectional (UD) CFRP straps are widely implemented as rigging systems in the sailing industry and particularly in racing sailboats [5,6]. They have also emerged in the crane industry as an alternative to steel pendant links in crawler cranes, allowing for improved lifting capacities and easier self-erecting assembly [7]. Furthermore, pin-loaded CFRP straps have been used successfully as tendons in the bowstring arch pedestrian bridge at the Swiss Federal Laboratories for Materials Testing and Research, Empa, Switzerland [8], and they have been recently proposed as deck suspenders in arch bridges [9]. In half-through and through arch bridges, the corrosion and fatigue of conventional steel suspenders is a critical factor that limits their design lifespan. Hence, there is a high potential in using corrosion- and fatigue-resistant pin-loaded CFRP straps for this purpose [9]. The current paper is aimed at studying the fatigue behaviour of these novel tensile elements in order to provide useful insights for the development of durable pin-loaded CFRP strap suspension systems.

In CFRP composites, the damage modes and crack propagation characteristics are considerably more complex compared to isotropic materials, such as metals, ceramics, or unreinforced polymers. They depend on a multitude of factors, such as the fibre and matrix types, the fibre sizing (thus, the fibre–matrix interaction), the layup sequence of the laminate, and the loading conditions, amongst many others [10,11,12,13]. A general characterisation of the fatigue behaviour of multiaxial fibre reinforced polymer composites (FRPs) was given by Reifsnider and Highsmith [14], who divided it into three different stages. In the first stage, damage develops at a very rapid rate within the first 10–15% of the life of the laminate, with the primary damage mode being cracking of the matrix in the laminae with the most off-axis fibre orientation. This intralaminar matrix cracking between the fibres reaches uniform saturation spacing at the end of stage I, which is called the characteristic damage state (CDS). In stage II, corresponding to 70–80% of the fatigue life, damage is still initiated and the existing damages continue to grow, although at a considerably slower rate, until the laminate is severely damaged. Finally, in stage III, the damage process is accelerated again until ultimate failure of the laminate.

In the case of purely unidirectional CFRP laminates where no off-axis plies are present, the only limiting factors in the fatigue life are the matrix and its interaction with the fibres [15,16,17] since carbon fibres show excellent fatigue behaviour [18]. Based on a review of the fatigue damage mechanisms, Talreja [19] introduced a fatigue life diagram in which he suggests that the fatigue life of a unidirectionally reinforced polymer is governed by the quasi-static fracture strain of the composite and the matrix fatigue limit strain. Given the insensitiveness of the fibres to fatigue and neglecting possible fretting, the diagram states that no damage progression takes place in the composite below the matrix fatigue limit strain (*ε_mf_*) [19], for which Talreja considered a value of *ε_mf_* = 0.6% based on experimental data for epoxy resins given by Dharan [20]. Hence, a matrix strain of 0.6% can be used as a minimum limit strain for epoxy fatigue when designing for the fatigue endurance limit.

When CFRP elements are in frictional contact with other components and subjected to relative cyclic displacement, their fatigue life can be drastically reduced due to the fretting process that occurs at the material interfaces. In general, the fretting wear performance of FRP laminates depends on many different factors, such as the fibre and matrix materials, the fibre orientation, the sliding direction, the contact pressure on the fretted surface, the amplitude and frequency of the reciprocating motion, the hardness of the contacting material, surface treatments, lubrication, and interfacial temperature conditions [21,22,23,24,25]. As regards the sliding direction specifically, the highest wear resistance occurs when sliding occurs parallel to the fibres, and the lowest for sliding perpendicular to the fibres [26,27,28]. In their pioneering study on the fretting fatigue behaviour of CFRP laminates, Schulte et al. [21,29,30] observed reductions up to three orders of magnitude in the fatigue life of laminates that were exposed to fretting on their 0° (load carrying) fibre plies, compared to those tested under plain fatigue. However, only slight deviations were observed in the fretting and plain fatigue behaviour of laminates if their outer plies comprised off-axis (±45°) fibres [21]. In the case of sliding parallel to the load-bearing longitudinal fibres, the effects of fretting wear are detrimental to the fatigue life of the laminate. On the other hand, for laminates comprising distinct off-axis fibres at the fretted surface, the fatigue life is comparatively less sensitive to the fretting component despite the higher wear rate of the outer ply as compared to sliding along longitudinal fibres. This is because by (sacrificially) damaging the outer off-axis plies, the underlying load-bearing longitudinal plies are protected [21].

These phenomena are highly relevant to the durability of CFRP straps in applications such as those described above, since they are subjected to fatigue loading while being in frictional contact with the pins. In a previous study by the authors [31], the fretting fatigue behaviour of laminated unidirectional CFRP straps as suspender models was investigated. The current paper studies the fretting fatigue behaviour of model straps (MS) comprising an additional sacrificial fibre ply between the innermost ply of the looped CFRP strap and the loading pin. Introducing a thin, low-stiffness/toughness fibre ply (e.g., glass: GFRP) between the innermost ply of the CFRP strap and the loading pin has been suggested by Schürmann [32] as a way to reduce the stress concentrations at the curved part of the strap. In addition to this function, such a ply is considered in the current paper as sacrificial protection against fretting of the load-bearing 0° fibres of the CFRP strap. Because a glass fibre reinforcement significantly reduces the fretting wear resistance of the composite laminates [22], a carbon fibre sacrificial ply was used instead. This comprised a woven fabric with fibres oriented in the ±45° direction, such that their influence on the axial stiffness of the strap is reduced, while still retaining significant resistance to fretting, as opposed to transversely oriented fibres [21]. Another expected advantage of using a fabric made of carbon was that a possible fretting product might be graphite, which is known to be a good lubricant under the right conditions [33].

Finally, in addition to the small-scale model straps, the behaviour of pristine full-scale laminated CFRP straps (FSS) in fretting fatigue tests without a sacrificial ply is also discussed.

## 2. Materials and Methods

### 2.1. Materials

The investigated CFRP straps were manufactured from out of autoclave (OOA) prepreg. The unidirectional tapes were manufactured by Carbo-Link AG in Fehraltorf, Switzerland, with widths of 12 ± 1 and 48 ± 1 mm for the model and full-scale straps, respectively, using intermediate modulus Tenax^®^ -J IMS60 carbon fibre rovings by Toho Tenax Europe GmbH, Wuppertal, Germany, [34] in combination with the hot melt epoxy matrix system XB3515/Aradur^®^ 5021 by Huntsman Advanced Materials GmbH, Basel, Switzerland, [35]. The two tapes contained 3 and 12 rovings, respectively, with 24,000 fibres per roving. The carbon fibres had a reported tensile strength of 5800 MPa and a Young’s modulus of 290 GPa [34], and the tape was reported to have a fibre volume content (*V_f_*) of 62 ± 2% and a weight of 290 g/m^2^ [36]. *V_f_* measurements by sulphuric acid digestion of the epoxy according to [37] revealed an average *V_f_* of 64.8%. The OOA prepreg used for the sacrificial ply of the model straps and for the confinement of the transition zone in the full-scale straps used the same matrix system as the unidirectional tape but consisted of HexTow^®^ AS4 carbon fibres by Hexcel Corporation, Stamford, CT, USA, with a reported Young’s modulus of 231 GPa and a tensile strength of 4619 MPa [38]. The fibres were weft in 2 × 2 twill style to a fabric with a grammage of 193 g/m^2^. The final prepreg grammage was reported to be 343 g/m^2^ [36].

The pins used for loading the small-scale model straps were pultruded T300 CFRP pins with a diameter of 20 mm and a reported *V_f_* of 60–65% [39]. For the loading of the full-scale straps, 60 mm wide titanium connector eyes with an inner and outer diameter of *d_i_* = 90 mm and *d_o_* = 200 mm were used. A 10 mm deep and 50 mm wide circumferential notch in the connector eyes ensured a proper positioning and lateral support of the laminate.

### 2.2. Manufacturing

**Figure 1:**
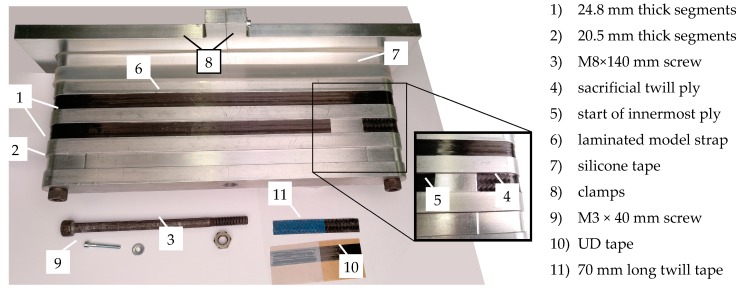
Mould for lamination of the model carbon fibre reinforced polymer (CFRP) straps with a sacrificial ply in the contact areas.

**Figure 2:**
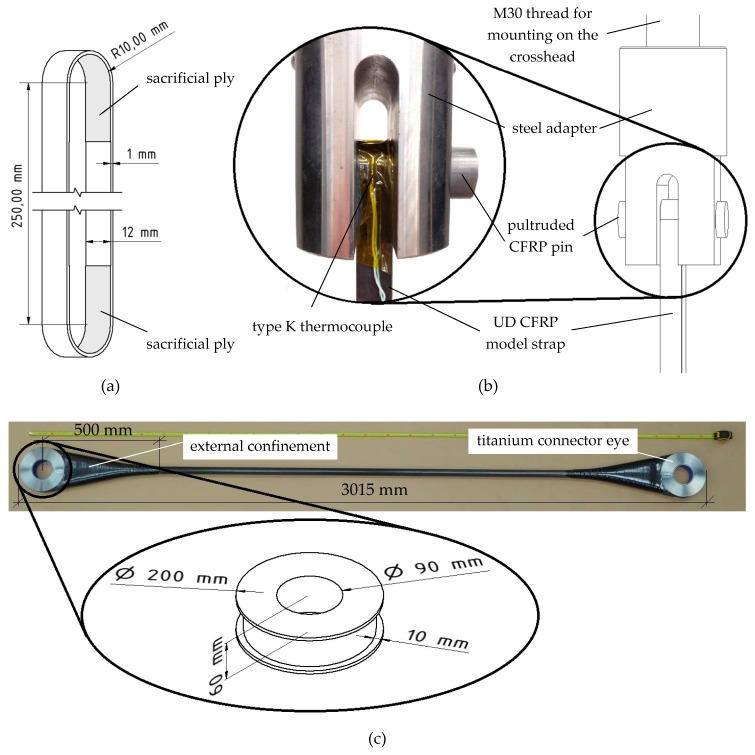
Investigated unidirectional CFRP straps: (**a**) dimensions of the model straps; (**b**) test setup of the model straps on the testing machine; (**c**) pristine full-scale strap before testing with detail of the titanium connector eye.

Both types of straps were manufactured using a winding process. The model straps were laminated in a similar process to that previously described in [31]. In order to accommodate the sacrificial plies in the curved (contact) areas without influencing the orientation of the unidirectional (load-carrying) fibres, the aluminium mould described in [31] had to be adjusted. Figure 1 illustrates how the thinner segments (grooves) of the mould, around which the tapes were wound, were thickened in the shaft region by 0.25 mm on each side (i.e., the thickness of the woven CFRP ply) such that the load-carrying unidirectional fibres were not bent during the lamination process. As an improvement to the lamination process described in [31], the laminate was compressed by two clamps that were screwed together in the shaft region of the straps. This was to ensure a well-defined laminate quality in critical (curved) regions of the model straps. The model straps had a nominal shaft length *L* of 250 mm, a nominal width and thickness of 12 mm and 1 mm, respectively, and an inner radius of curvature of 10 mm, see Figure 2a. The thickness of the laminate was chosen such that the ratio of outer to inner radius of curvature (a ratio of 1.1) was similar to that of the full-scale straps. This has been reported in the literature to be the governing factor for the tensile efficiency of laminated unidirectional FRP composite straps [40].

The full-scale straps were manufactured by winding the 48 mm wide tape directly around the two titanium connector eyes to a final thickness of 10 mm using a tape laying machine [41]. The transition zone between connector eyes and shaft was internally supported with proprietary foam [42] and externally confined with the woven twill fabric described above in order to restrain the mode I opening forces acting on the shaft under tensile loads. Then, shrinking tape was wound around the free length and transition zone of the straps in the circumferential direction. This ensured proper compaction of the laminate during the curing process and a circular cross-sectional area over the free length in the final full-scale straps. The three full-scale straps were denominated FSS A, B, and C, had a cross-sectional area of 871, 860, and 845 mm^2^ and a length of 3012, 3015, and 3017 mm, respectively. Figure 2c shows a full-scale strap before testing.

Both types of straps were cured at 140 °C for 2.5 h.

### 2.3. Experimental Setup

Figure 2b illustrates the test setup of a model strap and pin in the testing machine. The ultimate tensile strength and fretting fatigue tests on the model straps were conducted on a servo-hydraulic testing machine (type 1251, Instron^®^, High Wycombe, UK). The tensile strength tests were performed under displacement-control at a cross-head speed of 2 mm/min. The corresponding fatigue tests were performed under (peak-) load-control at a frequency *f* of 10 Hz and a load ratio *R* of 0.1. The pultruded CFRP pins were placed in fork-like steel adapters that are screwed to the crosshead and pulsator of the testing machine. Figure 2b also shows the type K thermocouple that was attached to the outside of the straps during the fatigue tests in order to monitor the temperature development in the critical (vertex) area. It was considered important to assess the temperature increases, e.g., from hysteretic heating at high test frequencies or due to fretting, because they can reduce the fatigue performance of a composite structure.

The full-scale straps were tested on an in-house testing machine containing an Amsler P960 pulsator. The titanium connectors of the full-scale straps were mounted on a metal bolt with an intermediate plain-bearing brush. The quasi-static tensile strength test of FSS C was performed under force-control at 90 kN/min. FSS A was fatigue-tested at a frequency of 4.2 Hz and *R* = 0.2 for 800,000 load cycles (*N*) and an additional 11.6 × 10^6^ load cycles at a load ratio of 0.42. The corresponding upper load levels (*F_u_*) were 575 and 462 kN, respectively. FSS B was fatigue-tested at a load ratio of 0.42 and a frequency of 4.2 Hz for 11.3 × 10^6^ load cycles at *F_u_* = 462 kN.

## 3. Results

Thickness measurements of the model straps revealed a high scatter originating from their manufacturing process. A measured width variation of ±1 mm in the prepreg tape led to local discontinuities in the overlapping of the individual plies during lamination, resulting in extra plies if the tape was too wide or lacking plies wherever the tape was less than 12 mm wide. The chosen clamping system was not able to cope with these discontinuities and compact the laminate evenly, which resulted in a standard deviation of 10% of the laminate thickness. Hence, there were comparatively large local thickness variations in the model straps that made it difficult to determine the exact cross-sectional area of the straps and thus also the acting stresses in the model straps. As all model straps were manufactured from the same 12 mm wide tape and all full-scale straps were manufactured from the same 48 mm wide tape, for a better comparison of the two strap types, the apparent fibre stress in the straps (*σ_afs_*) is introduced and used throughout this study. It is calculated as follows:

(1) $$\sigma_{afs}=\frac{F}{A_{f}}$$

with *F* being the load (in (N)) and *A_f_* the total cross-sectional area (in (mm^2^)) of the unidirectional fibres in the shaft area in the respective strap. Due to the same amount of plies in each of the respective straps, this cross-sectional area was constant at a value of 16.96 mm^2^ for the model straps and 531.56 mm^2^ for the full-scale straps. The fibres of the sacrificial twill ply are excluded, since they are only applied locally and do not contribute to the load-bearing capacity of the strap in the shaft areas. With a Young’s modulus ratio of approximately 1:100 between the epoxy matrix and the IMS60 fibres and a *V_f_* of 64.8%, the contribution of the matrix to the load-bearing capacity in the strap shafts is less than 0.5% and is therefore neglected as well.

### 3.1. Quasi-Static Behaviour

In order to quantify the performance of the investigated straps under cyclic fretting fatigue loads, their pristine load-carrying capacity under quasi-static tensile loads had to be assessed first. Hence, five model straps and one full-scale strap were subjected to quasi-static tensile tests until failure. Table 1 lists the strength and stiffness values obtained from these tests.

The similar straps of the previous study [31] that did not have a sacrificial ply were characterised by an average tensile strength of 1,624 ± 121 MPa. This corresponds to an apparent fibre stress of 2368 ± 205 MPa at an average maximum tensile load of 40.2 ± 3.4 kN. The reported Young’s Modulus of the straps in [31] of 175.8 ± 12.1 GPa corresponds to an apparent Young’s Modulus of 259.9 ± 15.2 GPa. Comparing the results in Table 1 with [31], it can be said that the model straps with an additional, sacrificial ply in the curved areas show marginally higher strength (+4.9%) and stiffness (+6.8%) values at marginally higher coefficients of variation (CoV: ratio of standard deviation to the mean value) compared to model straps without a sacrificial ply. The full-scale strap reached a significantly higher apparent ultimate tensile stress (*σ_afs,max_*) than that of the model straps (+36.9%).

### 3.2. Fretting Fatigue

**Figure 3:**
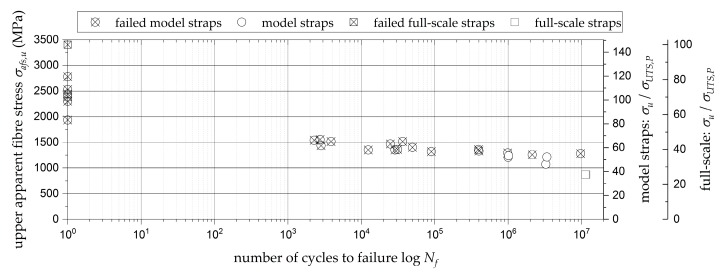
S-N curve of the model straps with a woven CFRP sacrificial ply (circular markers) and the full-scale straps (square markers). Listed are the upper apparent fibre stresses (*σ_afs,u_*) over the number of endured load cycles (*N_f_*). Data points containing a cross indicate failed straps.

Figure 3 shows the S-N curve obtained from the quasi-static and fretting fatigue testing of 25 pin-loaded model straps with a sacrificial ply (circular markers). As the pristine model straps with a sacrificial ply performed equal to the pristine straps tested in [31] under quasi-static loading, it was expected that they would perform even better under cyclic fretting fatigue loading. Therefore, the first fatigue tests were performed at an upper load level (*F_u_*) of 60% of the quasi-static tensile strength of the model straps. At this load level, the straps already failed prematurely at less than 10^4^ load cycles. In order to determine the fatigue limit load of the model straps with a sacrificial ply, *F_u_* was continuously reduced. At load levels below 55% of the pristine tensile strength of the model straps, the first straps endured more than 1 Million load cycles. However, out of those six straps, only four did so without failure. Two of the straps tested for *N* >10^6^, at 52.5% and 53.5% of the pristine *F_max_*, failed after 2.1 and 9.7 million load cycles, respectively.

Although the full-scale straps were tested at a different frequency and load ratio (*f* = 4.2 Hz, *R* = 0.2, 0.42) than the model straps, the results from the full-scale strap tests (square markers) are also included in Figure 3. Both full-scale straps tested at 25.5% of the pristine full-scale strap tensile strength endured more than 11 × 10^6^ load cycles without failure (see also Table 2).

### 3.3. Residual Mechanical Properties

The residual mechanical properties of the straps that did not fail under cyclic fretting fatigue loading were assessed in quasi-static tensile tests as described in Section 2.3. Table 3 lists the results of these tests.

The comparison of the residual mechanical properties to the mechanical properties of pristine straps (Table 1) shows that the ultimate tensile strength of the model straps remained almost unchanged. At the same time, their apparent fibre parallel stiffness decreased by 10.4%. This might be attributed to damages observed in the fatigued laminate, i.e., delaminations and fibre-parallel matrix cracks that increase the longitudinal compliance of the laminate, but only marginally affect its ultimate tensile strength. Similar results have also been presented in [31]. The strains on the full-scale straps could not be measured, but the test revealed a significant decrease in ultimate tensile strength of the full-scale straps after a cyclic fretting fatigue loading of 25%. This much stronger strength decrease is in good agreement with the reported stronger reduction in fatigue life when using a harder pin material in contact with a unidirectional CFRP ply loaded in the fibre direction [29].

### 3.4. Damage Modes

**Figure 4:**
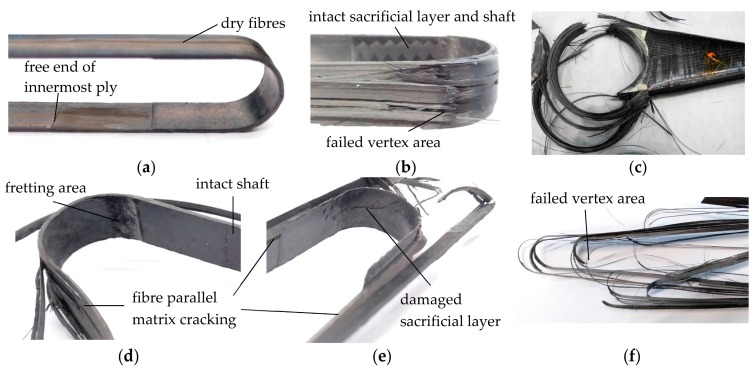
Damage modes in different fretting-fatigue-loaded model straps with sacrificial twill plies: (**a**) intact model strap prior to testing, showing the sacrificial ply and the free end of the innermost UD-ply; (**b**) failed model strap (*N_f_* = 9.7 × 10^6^) with intact sacrificial ply; (**c**) failed full-scale strap FSS C with failure in the vertex area; (**d**) failed model strap (*N_f_* = 2.1 × 10^6^) with intact sacrificial ply; (**e**) failed model strap with damaged and delaminated sacrificial ply (*N_f_* = 9 × 10^4^); (**f**) failed model strap after residual tensile strength test (*N* = 3.7 × 10^4^).

All model straps included in this study initially failed in their vertex areas (see Figure 4b) with subsequent fibre bursting in the shaft or crown area. The first visible damages of the model straps were delamination of the inner and outermost plies in the shaft with the overlapping plies. Delaminations initiated at the free ends of the overlaps and progressed towards the vertex areas where they stopped. The same fibre-parallel longitudinal cracking as described in [31] was observed on the model straps with a sacrificial twill ply during the fatigue tests prior to failure. After the premature failure in the vertex area of the straps due to the cyclic fretting fatigue loading, different types of secondary failures were observed. Figure 4d shows a failed strap with the inner part of the curved section and one shaft (right) being still mostly intact, whilst the outermost plies and the opposite shaft (left) are severely damaged. Figure 4e shows a strap where both shafts and the complete curved section, including the sacrificial ply, were severely damaged. Figure 4b,d,e are representative images of all observed premature failures during cyclic fretting fatigue loading of the model straps. However, there appears to be no correlation between the different types of secondary failure and the (upper) load level that the straps were exposed to during testing. Figure 4f shows a model strap after failure due to a residual ultimate tensile strength test. All model straps tested for their pristine or residual ultimate tensile strength failed primarily in the vertex area with a more pronounced secondary fibre bursting in the shafts compared to the prematurely failed fatigue-tested straps. In case of the model straps tested for their residual ultimate tensile strength, this fibre bursting in the shaft resulted in more and smaller bristles due to the previous fibre-parallel damaging of the laminate.

The failure of full-scale strap FSS C occurred similar to the model straps, with initial failure in the vertex area (see Figure 4c) followed by fibre bursting in the shaft. The fatigue-tested full-scale straps showed no signs of fibre bursting in the shaft but significant fibre-parallel matrix cracking.

### 3.5. Fretting Behaviour of Contacting Surfaces

**Figure 5:**
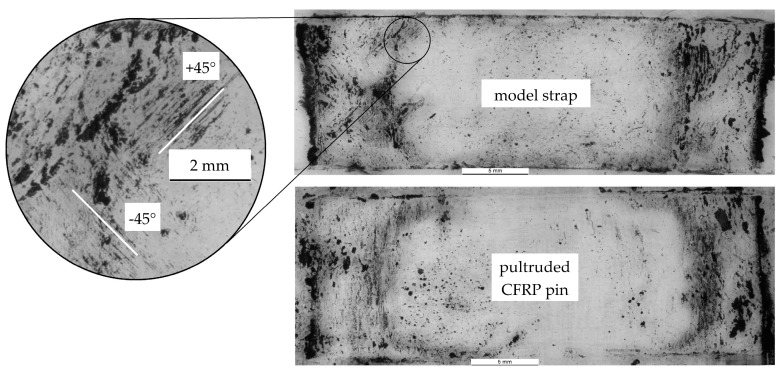
Fretting products of a model strap with a sacrificial twill ply (top) and a pin (bottom) after *N* = 3.37 × 10^6^. Tested at an upper load level of 20.596 kN (*σ_afs,u_* = 1214 MPa).

The fretting behaviour of the contacting surfaces was investigated differently for the two types of straps. By placing a transparent adhesive tape on the model strap and CFRP pin surfaces just after testing, the fretting products of these tests could be investigated. The tapes were placed on microscope slides and were examined under an optical microscope (ZEISS Stemi SV 11 in reflected-light mode). Figure 5 shows two representative pictures of the fretting products on a strap and a pin. The fretting products on the pin look similar to those described in [31], with mostly short, broken fibres and resin particles still attached to them. The fretting products on the strap are mainly small carbon particles (see [31]). However, the presence of the contacting twill ply can be clearly seen from the fibres oriented in ±45° (relative to the unidirectional fibres along the strap axis). However, these ±45° fibres were only visible on one of the two fretting areas.

**Figure 6:**
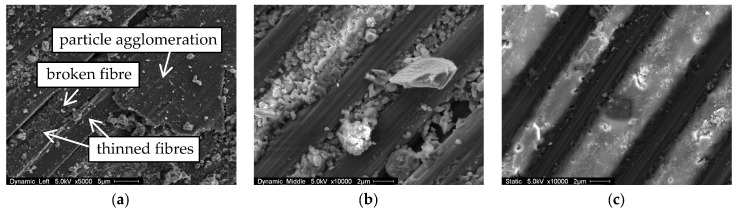
Typical SEM images of the full-scale strap surfaces in contact with the titanium connector eye. The images were taken after failure of the straps: (**a**) in the vertex area of FSS A; (**b**) close to the top (crown) area of FSS A; (**c**) in the vertex area of FSS C.

The contacting surfaces of the full-scale straps were investigated under a scanning electron microscope (SEM, FEI ESEM XL30). Figure 6 shows three pictures of typical surface conditions after testing. Figure 6c shows the surface of FSS C in contact with the titanium connector eye in the vertex area. As the strap was not exposed to fatigue testing, the fibres (dark) are still well-embedded in the intact matrix (light) and show no signs of fibre thinning. In the regions close to the top (crown) where the relative movement between strap and connector eye is relatively small, the surface of the fatigue tested FSS A was similar. Further away from the top (crown) of the strap, see Figure 6b, the fibres are still intact, but the matrix suffered from the small relative movement between the strap and the connector eye. This matrix deterioration is even more pronounced in the vertex areas of fatigue tested straps, Figure 6a, where the fibres are not visibly embedded in the matrix anymore. The fibre-parallel fretting also caused fibre thinning (Figure 6a, left), and the fretting products agglomerated and covered the fibres (Figure 6a, right). These particle agglomerations consist of compressed fretting products with particle diameters of 30–100 nm.

### 3.6. Temperature

**Figure 7:**
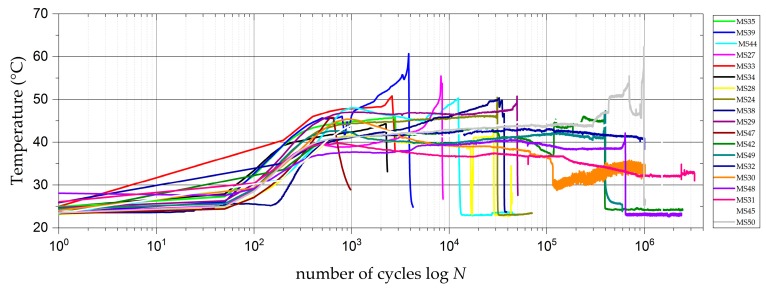
Temperature development on the outside of the fretting-fatigue-tested model straps with a sacrificial ply.

Due to the high testing frequencies, the temperature on the outside of the straps was monitored during all fatigue tests. The thermocouples on the outside of the model straps measured a significant initial temperature increase, which reached a peak value within the first 5000 load cycles. In contrast to straps tested without a sacrificial ply [31], the temperature does not drop off significantly after this peak, but levels out at an only a slightly lower temperature (see Figure 7). The temperatures increase again once final failure of the straps is initialised. The maximum measured initial temperature peaks on the outside of the model straps were all below 50 °C. With a reported glass transition temperature (*Tg*) of the epoxy matrix system of 140 °C [35], these temperatures never endangered the structural integrity of the straps.

The temperature measurements on the outside of the full-scale strap FSS A revealed a maximum temperature of 80 °C after 2 × 10^4^ load cycles. As the temperature did not decrease within the subsequent 5000 load cycles and with a laminate thickness of 10 mm, it had to be assumed that the temperature inside the laminate was much higher. Consequently, the test was paused and *F_u_* was reduced from 575 kN (*σ_afs,u_* = 1081 MPa) to 462 kN (*σ_afs,u_* = 869 MPa) to ensure the laminate temperature did not exceed *Tg* anywhere in the laminate. After these adjustments, the temperature did not exceed 50 °C, which was also the maximum temperature measured on FSS B during the cyclic fretting fatigue loading.

## 4. Discussion

The laminated unidirectional CFRP tension members investigated in this study were shown to be a good alternative to well-established steel tension members in terms of their fretting fatigue behaviour [44,45,46]. The introduction of an additional sacrificial ply protecting the load-carrying fibres during the cyclic fretting fatigue tests did not result in the expected increase in durability of the investigated model straps as the sacrificial plies did not influence the failure mode. The failure was still initiated and determined by the fibre-parallel stress concentrations in the vertex areas of the straps as described in [31]. One strap failed only after 9.7 × 10^6^ load cycles, which is far above 3 ×10^6^ load cycles. This has previously been reported to correspond to the fretting fatigue endurance limit of comparable straps [31]. However, complications with proper compaction of the model strap laminate during curing resulted in strongly varying thicknesses of the straps along their width. Although the laminate quality could be shown to be satisfying in general (*V_f_* = 64% and a void content of 1%), such surface irregularities lead to stress concentrations and can act as stress inducers that have a strong influence on the fatigue behaviour of CFRP laminates [47] and should hence be eliminated in future studies.

The ultimate tensile strength tests of pristine straps revealed significantly higher failure stresses in the full-scale straps. A better laminate quality due to the robotic manufacturing of the straps with a tape-laying machine, the different pin geometry, and the lateral confinement of the full-scale strap, which reduced unfavorable stress concentrations in the transition zone, might, however, explain these results. The residual ultimate tensile strength tests of both the model and full-scale straps resulted in similar apparent failure stresses of both strap types at around 2500 MPa. This in turn means that the full-scale strap strength was reduced by 25% due to the fretting fatigue testing, whilst the model strap strength remained almost the same. The strong reduction of the full-scale strap strength can be tentatively attributed to the harder pin material and the larger contacting area (see [29]) acting on the laminate. The average residual failure stresses of the model straps on the other hand are almost the same (100.3%) as the pristine tensile strength of model straps. This supports the findings in [31], where the residual strength of the straps corresponded to 99.7% of the pristine tensile strength.

The temperature measurements on the model straps showed no perturbing temperature increase due to the cyclic fretting fatigue loading. The different geometry, a thicker laminate, and different material partners in combination with a larger contacting area led to a strong temperature increase on the full-scale strap surface that was too high to disregard, and adequate measures had to be taken to inhibit the high temperatures.

The presented results are part of a pioneering study on the fretting fatigue of fibre-dominated tensile elements. The model straps used throughout this study had a similar ratio of outer to inner radius of curvature as the full-scale straps. Nevertheless, the different quasi-static strengths and the different fretting behaviour (e.g., with respect to temperature development and residual properties) of the two strap types have shown that further studies are necessary. Such studies should focus on size effects, e.g., by upscaling the model strap without lateral confinement or variation of the radius of curvature ratio, as well as on the influence of the connector eye material and geometry on the fretting fatigue behaviour. Furthermore, the fact that the ±45° fibres were only observed in one fretting area should be investigated as it might be a result of an uneven loading that could result in an additional reduction of the fatigue resistance of the straps. Future research should also contemplate the behaviour of pin-loaded CFRP straps exposed to harsh environmental conditions, such as salt water, humidity, and high temperatures.

## Figures and Tables

**Table 1:** Quasi-static tensile strength and stiffness values of one full-scale strap and five model straps. The apparent fibre parallel elastic modulus *E_11,af_* (GPa) was calculated from linear encoder measurements following [43] using the respective fibre cross-sectional areas (*A_f_*). The apparent ultimate tensile stresses (*σ_afs,max_*) and loads (*F_max_*) are also given.

Strap	*F_max_* (kN)	*σ_afs,max_* (MPa)	*E_11,af_* (GPa)
MS23	47.171	2781	262.614
MS25	41.441	2443	297.391
MS37	39.009	2299	248.343
MS40	42.888	2528	278.469
MS41	40.447	2384	301.277
Ø	42.2 ± 3.1	2485 ± 185	277.619 ± 22.5
FSS C	1809	3403	-

**Table 2:** Summary of all fretting fatigue tests conducted in this study. Given are the absolute upper load *F_u_* (kN), the apparent upper fibre stress *σ_afs,u_* (MPa), the number of tested cycles *N*, the corresponding load ratio *R*, the testing frequency *f* (Hz), and the information on whether the respective strap suffered from premature failure or not.

Strap	*F_u_* (kN)	*σ_afs,u_* (MPa)	Tested cycles *N* (-)	Load ratio *R* (-)	Frequency *f* (Hz)	Failure
MS34	26.106	1539	2276	0.1	10	yes
MS33	26.373	1555	2737	0.1	10	yes
MS35	24.360	1436	2823	0.1	10	yes
MS39	25.684	1514	3864	0.1	10	yes
MS44	22.927	1351	12,423	0.1	10	yes
MS27	24.900	1468	24,900	0.1	10	yes
MS28	22.931	1352	28,755	0.1	10	yes
MS24	23.065	1360	31,300	0.1	10	yes
MS38	25.684	1514	36,606	0.1	10	yes
MS29	23.780	1402	49,729	0.1	10	yes
MS47	22.376	1319	90,007	0.1	10	yes
MS42	23.084	1361	394,770	0.1	10	yes
MS49	22.563	1330	401,058	0.1	10	yes
MS50	21.955	1294	989,067	0.1	10	yes
MS32	20.485	1208	1,002,100	0.1	10	yes
MS30	21.018	1239	1,015,490	0.1	10	no
MS48	21.336	1258	2,129,280	0.1	10	yes
MS31	18.202	1073	3,267,560	0.1	10	no
MS46	20.596	1214	3,373,240	0.1	10	no
MS45	21.720	1280	9,723,500	0.1	10	yes
FSS A	575	1082	800,000	0.2	4.2	no
	462	869	11,600,000	0.42		
FSS B	462	869	11,300,000	0.42	4.2	no

**Table 3:** Residual mechanical properties of the straps tested without failure. Given are the upper load level during the cyclic fretting fatigue tests (***σ_afs,u_***) in (MPa), the number of cycles tested (*N*), the maximum residual tensile load carrying capacity (*F_r,max_*) in (kN) and (%) of *F_max_*, the apparent maximum residual tensile stress (*σ_r,afs,max_*) in (MPa) and the apparent residual fibre parallel Young’s Modulus (*E_r11,af_*) in (GPa) and (%) of *E_11,af_*.

Strap	*σ_afs,u_*	*N*	*F_r,max_*		*σ_r,afs,max_*	*E_r11,af_*	
	(MPa)		(kN)	(%)	(MPa)	(GPa)	(%)
MS30	1239	1,015,494	40.878	96.95	2410	252.407	90.92
MS31	1073	3,267,564	42.766	101.43	2521	240.496	86.63
MS32	1208	1,002,102	43.049	102.10	2538	-	-
MS46	1214	3,373,241	42.526	100.86	2507	253.425	91.29
Ø	1183 ± 75	-	42.3 ± 0.98	100.3 ± 2.3	2494 ± 57	248.8 ± 7	89.6 ± 2.6
FSS A	869	11,600,000	1302.48	72.00	2450	-	-
FSS B	869	11,300,000	1411.02	78.00	2655	-	-
Ø	869 ± 0	-	1357 ± 77	75.0 ± 4.2	2553 ± 145	-	-
